# LINC01342 silencing upregulates microRNA-508-5p to inhibit progression of lung cancer by reducing cysteine-rich secretory protein 3

**DOI:** 10.1038/s41420-021-00613-x

**Published:** 2021-09-09

**Authors:** Qiming Shen, Zhe Xu, Guanghao Sun, Haoyou Wang, Lin Zhang

**Affiliations:** Department of Thoracic Surgery, The First Hospital of China Medical University, 110001 Shenyang, Liaoning China

**Keywords:** Diseases, Cell biology

## Abstract

Long noncoding RNAs (lncRNAs) are critical players during cancer progression. Nevertheless, the effect of most lncRNAs in lung cancer (LC) remains unclear. We aimed to explore the role of LINC01342 in LC development through the microRNA-508-5p (miR-508-5p)/cysteine-rich secretory protein 3 (CRISP3) axis. LINC01342, miR-508-5p, and CRISP3 expression in clinical samples and cell lines were determined, and their correlations in LC were analyzed. The prognostic role of LINC01342 in LC patients was evaluated. LC cells were screened and, respectively, transfected to alter the expression of LINC01342, miR-508-5p, and CRISP3. Then, proliferation, migration, invasion, and apoptosis of transfected LC cells were determined, and the in vivo tumor growth was observed as well. Binding relationships between LINC01342 and miR-508-5p, and between miR-508-5p and CRISP3 were identified. LINC01342 and CRISP3 were upregulated and miR-508-5p was downregulated in LC tissues and cells. High LINC01342 expression indicated a poor prognosis of LC patients. The LINC01342/CRISP3 silencing or miR-508-5p elevation inhibited proliferation, migration, and invasion of LC cells and promoted LC cell apoptosis, and also suppressed the in vivo tumor growth. LINC01342 bound to miR-508-5p and miR-508-5p targeted CRISP3. LINC01342 plays a prognostic role in LC and LINC01342 silencing upregulates miR-508-5p to inhibit the progression of LC by reducing CRISP3.

## Introduction

Lung cancer (LC) is the most common malignancy in most countries and is still a leading cause of cancer-related death throughout the world. The high mortality of LC has resulted from the lack of LC screening and the late diagnosis caused by lacked physical symptoms in the early stages of LC [1, 2]. Traditionally LC is classified into two major histological types: non-small cell lung cancer (NSCLC) (75–85%) and small cell lung cancer (SCLC) (15–25%) [3]. The pathogenesis of LC is multifactorial, including genetic factors, environmental factors, and smoking, while LC occurrence is associated with the regulation of oncogenes and tumor suppressor genes [4]. It is reported that about 70% of LC patients were diagnosed at an advanced stage, with a 16% 5-year survival rate, and unfortunately, only 15% of LC patients were diagnosed at an early stage [5]. Thus, there is an urgency to explore the potential biomarkers for LC diagnosis and prognosis.

Long non-coding RNAs (lncRNAs) are transcripts with >200 nt in length whereas lack protein-coding ability. LncRNAs modulate various biological processes, such as cell viability, apoptosis, tumorigenesis, and immune response [6]. The involvement of several lncRNAs has been identified in LC. For instance, LL22NC03-N64E9.1 has been revealed to promote the proliferation of LC cells and play a prognostic biomarker for LC patients [7]. A study has demonstrated that lncRNA MNX1-AS1 facilitated LC cell growth, thus contributing to LC progression [8]. LINC01342 is a newly found lncRNA that remains scarcely studied. It has been elucidated that LINC01342 promoted the development of ovarian cancer (OC) [9]. Inspired by this study, here we speculated that LINC01342 may participate in other human cancers, such as LC. Moreover, through the bioinformatic prediction, we found that there existed a binding region between LINC0134 and microRNA (miRNA/miR)-508-5p. MiRNAs are small non-coding RNAs (~22 nt) that bind to the 3′-untranslated region (3′UTR) of target mRNAs, and function as post-transcriptional regulators of mRNA expression [10]. As previously reported, miR-431 suppressed proliferation and metastasis of LC [11], and miR-451a attenuated doxorubicin resistance in LC through repressing epithelial–mesenchymal transition [12]. As one of the miRNAs, miR-508-5p has been revealed to serve as a tumor repressor, which inhibited the growth of melanoma cells [13] and colorectal cancer (CRC) [14]. However, the role of miR-508-5p in LC remains largely unknown. Additionally, we also found the binding sites between miR-508-5p and cysteine-rich secretory protein 3 (CRISP3) on a bioinformatics website. CRISP3 was originally described as an androgen-dependent protein in mouse lacrimal and salivary glands [15]. In recent research, it was reported to drive the invasion and progression of prostate cancer [16]. Interestingly, another literature has shown that CRISP3 was upregulated in NSCLC patients after chemotherapy, validated using the microarray [17].

We performed this study to reveal the role of the LINC01342/miR-508-5p/CRISP3 axis in LC, and we speculated that LINC01342 may bind to miR-508-5p, which targets CRISP3 to affect the biological functions of LC cells, thus affecting LC development.

## Results

### LINC01342 is upregulated in LC tissues and cells, and the upregulation indicates a poor prognosis of LC patients

**Fig. 1:**
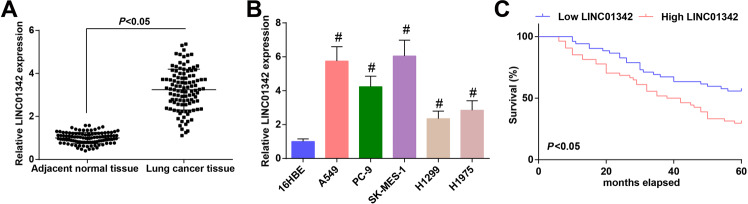
LINC01342 is upregulated in LC tissues and cells, and the upregulation indicates a poor prognosis of LC patients. **A** and **B** LINC01342 expression in clinical samples and cell lines was determined using RT-qPCR. **C** the prognostic role of LINC01342 in LC patients was detected using Kaplan–Meier analysis; #*P* < 0.05 vs. 16HBE cells.

At present, there are few reports about the clinical status, biological activity, or molecular mechanism of LINC01342 in LC. It was only found that LINC01342 was upregulated in OC [9]. In order to explore the expression of LINC01342 in LC tissues, we first compared the expression of LINC01342 in 106 pairs of LC tissues and adjacent normal tissues. It was discovered that the expression of LINC01342 in LC tissues was higher than that in adjacent normal tissues (Fig. 1A). Meanwhile, RT-qPCR showed that LINC01342 expression was increased in LC cell lines versus 16HBE cells, especially in A549 and SK-MES-1 cells (Fig. 1B).

To better understand the clinical significance of LINC01342 in LC, the 106 LC patients were divided into high expression group (*n* = 54) and low-expression group (*n* = 52) considering the expression of LINC01342 in all LC samples. Chi-square test showed that expression of LINC01342 was correlated with tumor-node-metastasis (TNM) stage (*P* = 0.034) and lymph node metastasis (LNM) (*P* = 0.025) (Table 1). To further study the effect of LINC01342 on the prognosis of LC patients, Kaplan–Meier analysis was performed to detect the survival of patients, and it showed that the overall survival of LC patients with high LINC01342 expression group was evidently shortened (Fig. 1C).

**Table 1:** Relationship between LINC01342 expression and clinicopathological characteristics of lung cancer patients.

Parameter	Case	LINC01342		*P* value
		Low expression (*n* = 52)	High expression (*n* = 54)	
*Age (years)*				0.567
<60	54	28	26	
≥60	52	24	28	
*Gender*				0.686
Male	68	32	36	
Female	38	20	18	
*Smoking history*				0.846
Yes	57	27	30	
No	49	25	24	
*Tumor size*				0.340
≤3 cm	54	29	25	
>3 cm	52	23	29	
*Histology type*				0.234
Adenocarinoma	64	28	36	
Squamous	42	24	18	
*Lymph nodes metastasis*				0.034
No	74	31	43	
Yes	32	21	11	
*TNM stage*				0.025
I–II	69	28	41	
III–IV	37	24	13	

The enumeration data were analyzed using the chi-square test.

*TNM* tumor-node-metastasis.

These findings revealed that LINC01342 was upregulated in LC tissues and cells, and this upregulation indicated a poor prognosis of LC patients.

### LINC01342 silencing inhibits malignant behaviors of LC cells in vivo and in vitro

**Fig. 2:**
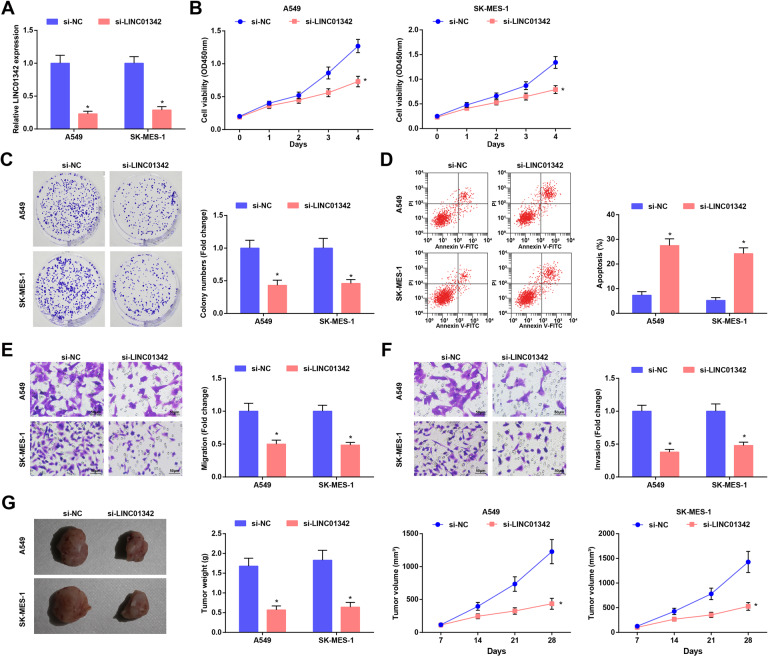
LINC01342 silencing inhibits malignant behaviors of LC cells in vivo and in vitro. **A** LINC01342 expression in LC cells was determined using RT-qPCR. **B** Cell viability was assessed using CCK-8 assay. **C** Colony formation ability of cells was detected using cell colony formation assay. **D** Apoptosis of cells was assessed using flow cytometry. **E** and **F** Migration, and invasion of cells was detected using Transwell assay. **G** Representative image, volume and weight of xenografts from nude mice; *P < 0.05 vs. the si-NC group.

In order to further explore the effect of LINC01342 on the biological characteristics of LC cells, we selected A549 cells and SK-MES-1 cells with high LINC01342 expression. Cells were transfected with si-NC and si-LINC01342, and RT-qPCR revealed that LINC01342 was successfully downregulated (Fig. 2A). CCK-8 results showed that the downregulation of LINC01342 inhibited the proliferation of A549 and SK-MES-1 cells (Fig. 2B). The colony formation ability of cells was detected using colony formation assay, and we found that LINC01342 silencing decreased the colony formation ability of cells (Fig. 2C).

The cell apoptosis was determined using flow cytometry and the data suggested that LINC01342 reduction promoted the apoptosis of A549 and SK-MES-1 cells (Fig. 2D).

Meanwhile, Transwell assay results showed that LINC01342 knockdown reduced the migration and invasion of A549 and SK-MES-1 cells (Fig. 2E, F).

In order to determine the effect of LINC01342 on the growth of LC cells in vivo, the xenotransplantation model was established by injecting cells stably expressing si-LINC01342 or si-NC into nude mice. The results showed that the tumor volume and weight were both suppressed after LINC01342 downregulation (Fig. 2G).

These results indicated that silencing LINC01342 inhibited the malignant behaviors of LC cells in vivo and in vitro.

### LINC01342 binds to miR-508-5p

**Fig. 3:**
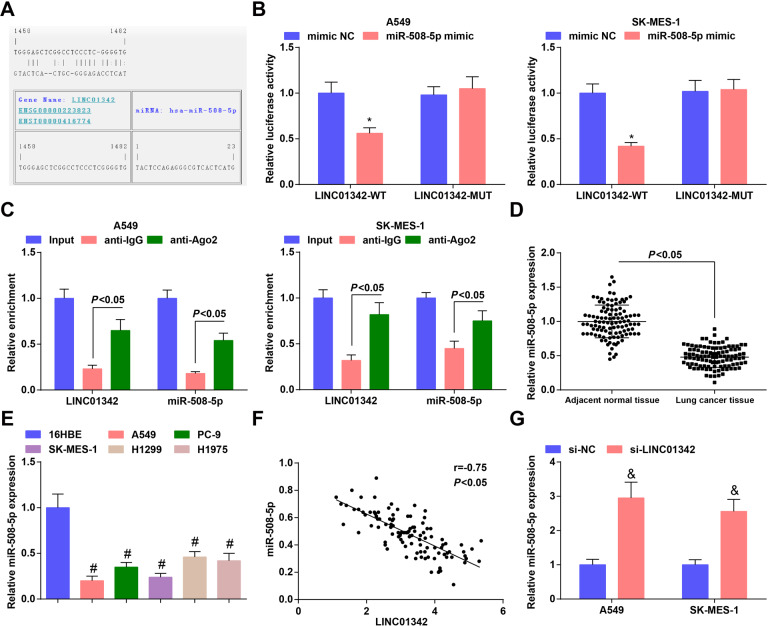
LINC01342 binds to miR-508-5p and regulates its expression. **A** Binding site between LINC01342 and miR-508-5p was predicted at RNA22. **B** Binding relationship between LINC01342 and miR-508-5p was confirmed using dual-luciferase reporter gene assay. **C** Enrichment of LINC01342 and miR-508-5p was detected using RIP assay. **D** miR-508-5p expression in LC tissues was determined using RT-qPCR. **E** miR-508-5p expression in LC cells was determined using RT-qPCR. **F** correlation between expression of LINC01342 and miR-508-5p was analyzed using the Pearson test. **G** miR-508-5p expression after LINC01342 silencing was determined using RT-qPCR. *P < 0.05 vs. the mimic NC group; #*P* < 0.05 vs. 16HBE cells, &*P* < 0.05 vs. the si-NC group.

A common mechanism of lncRNA activity is to act as bait or sponge for miRNA. In order to study the specific molecular mechanism of the biological effect of LINC01342, we predicted using the RNA22 website that there existed binding sites between LINC01342 and miR-508-5p (Fig. 3A).

To determine whether LINC01342 directly regulates miR-508-5p, we cloned the WT or MUT binding sequence of LINC01342 into luciferase reporter vector, and co-transfected it with miR-508-5p mimic or mimic NC into A549 and SK-MES-1 cells. The results showed that miR-508-5p mimics specifically inhibited WT-driven luciferase activity in A549 and SK-MES-1 cells, while did not affect LINC01342-MUT (Fig. 3B). In addition, the RIP assay revealed that LINC01342 and miR-508-5p were more abundant in Ago2 immunoprecipitation (Fig. 3C). The findings suggest that LINC01342 can specifically bind to miR-508-5p.

Next, we compared the expression of miR-508-5p between LC tissues and adjacent normal tissues, and the results showed that the expression of miR-508-5p was reduced in LC tissues (Fig. 3D, E). Pearson test showed that there was a negative correlation between expression of LINC01342 and miR-508-5p in LC tissues (Fig. 3F). To examine the regulation between LINC01342 and miR-508-5p, we used RT-qPCR to detect the expression of miR-508-5p after silencing LINC01342, and we found that miR-508-5p was upregulated in A549 cells and SK-MES-1 cells by LINC01342 reduction (Fig. 3G).

### Elevated miR-508-5p restricts malignant behaviors of LC cells

**Fig. 4:**
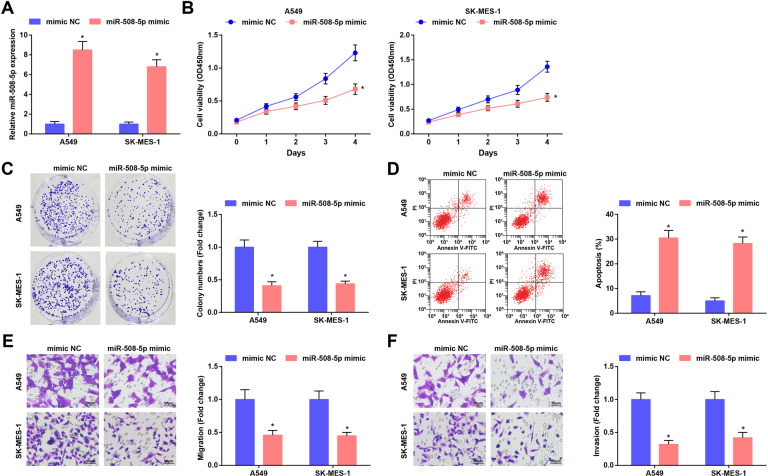
Elevated miR-508-5p restricts malignant behaviors of LC cells. **A** miR-508-5p expression in LC cells was determined using RT-qPCR. **B** Cell viability was assessed using CCK-8 assay. **C** Colony formation ability of cells was detected using cell colony formation assay. **D** Apoptosis of cells was assessed using flow cytometry. **E** and **F** Migration and invasion of cells were detected using Transwell assay; *P < 0.05 vs. the mimic NC group.

In order to understand the biological significance of miR-508-5p in LC, LC cells were transfected with miR-508-5p mimic to alter the miR-508-5p expression, and RT-qPCR showed that the transfection was successful (Fig. 4A).

After transfection, the biological functions of LC cells were determined, and it was found through CCK-8 assay, colony formation assay, flow cytometry, and Transwell assay that the miR-508-5p elevation inhibited the proliferation, colony formation ability, migration and invasion of LC cells, and also promoted LC cell apoptosis (Fig. 4B–F).

The above data implied that miR-508-5p upregulation suppressed the malignant phenotypes of LC cells.

### MiR-508-5p directly targets CRISP3

**Fig. 5:**
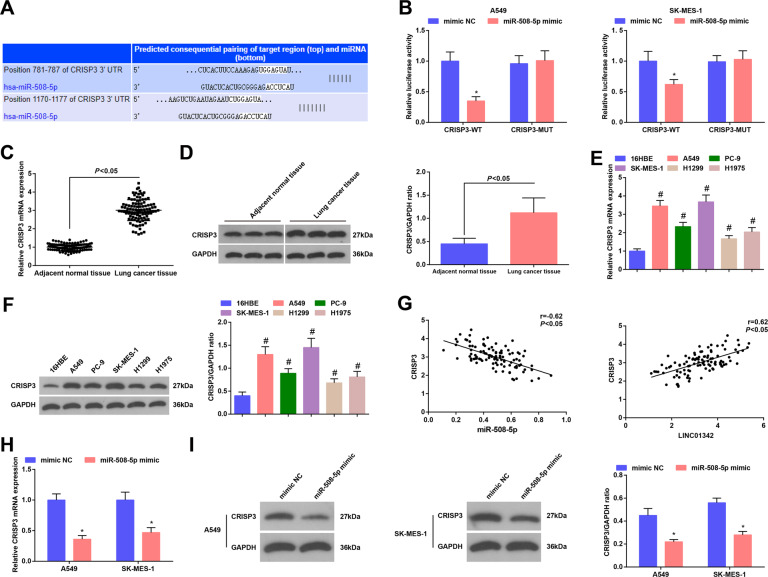
MiR-508-5p directly targets CRISP3. **A** Binding site between miR-508-5p and CRISP3 was predicted at Targetscan. **B** Targeting relationship between miR-508-5p and CRISP3 was confirmed using dual luciferase reporter gene assay. **C** mRNA expression of CRISP3 in LC tissues was determined using RT-qPCR. **D** Protein expression of CRISP3 in LC tissues was determined using Western blot analysis. **E** mRNA expression of CRISP3 in LC cells was determined using RT-qPCR. **F** Protein expression of CRISP3 in LC cells was determined using Western blot analysis. **G** Correlations between miR-508-5p and CRISP3, and between LINC01342 and CRISP3 were analyzed using Pearson test. **H** CRISP3 mRNA expression after miR-508-5p upregulation was detected using RT-qPCR. **I** CRISP3 protein expression after miR-508-5p upregulation was detected using Western blot analysis; *P < 0.05 vs. the mimic NC group; #*P* < 0.05 vs. 16HBE cells.

CRISP3 belongs to a cysteine-rich secretory protein family and has been revealed to be upregulated in prostate cancer [18]. We set out to examine whether CRISP3 is also a target of miR-508-5p in LC. First, targetscan analysis revealed that there are potential binding sites that may be targeted by miR-508-5p in the CRISP3 gene (Fig. 5A). Dual-luciferase reporter gene assay showed that miR-508-5p mimic specifically inhibited the expression of luciferase gene containing WT, but did not inhibit the CRISP3-MUT-binding site (Fig. 5B). Next, by examining the expression of CRISP3 in clinical samples and cell lines, we found that CRISP3 mRNA and protein expression was increased in LC tissues and cell lines (Fig. 5C–F). Correlation analysis showed that CRISP3 expression was negatively correlated with miR-508-5p expression, but was positively correlated with LINC01342 expression (Fig. 5G). In A549 and SK-MES-1 cells, upregulation of miR-508-5p inhibited CRISP3 mRNA and protein expression (Fig. 5H, I).

To further verify whether LINC01342 affects the expression of CRISP3, we silenced LINC01342 in A549 and SK-MES-1 cells and found that the mRNA and protein expression levels of CRISP3 were down-regulated (Supplementary Fig. 1).

These data suggest that miR-503-5p regulates CRISP3 expression directly by targeting CRISP3.

### CRISP3 reduction suppresses malignancy of LC cells

**Fig. 6:**
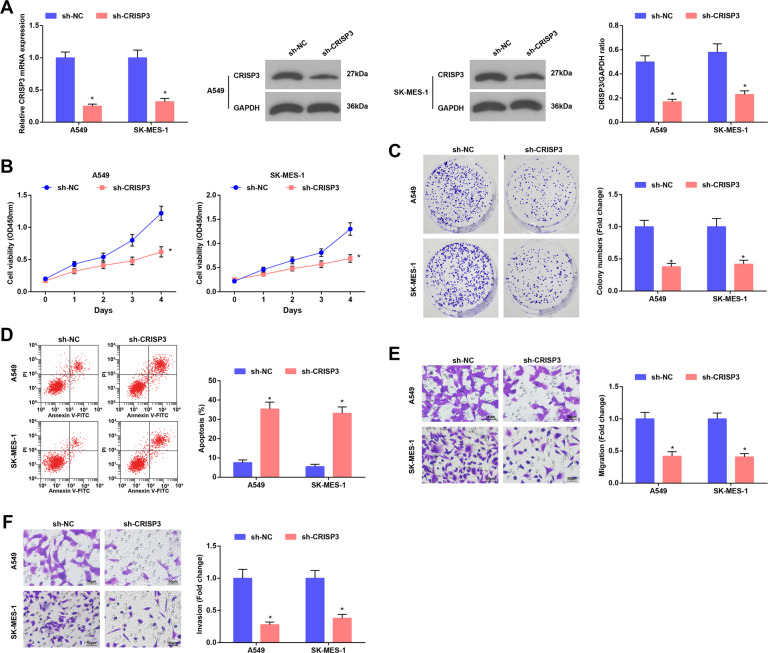
CRISP3 reduction suppresses the malignancy of LC cells. **A** CRISP3 mRNA and protein expression in LC cells was detected using RT-qPCR and Western blot analysis. **B** Cell viability was assessed using CCK-8 assay. **C** Colony formation ability of cells was detected using cell colony formation assay. **D** apoptosis of cells was assessed using flow cytometry. **E** and **F** Migration and invasion of cells was detected using Transwell assay; *P < 0.05 vs. the sh-NC group.

Cells were transfected with sh-CRISP3 to change CRISP3 expression, and it was confirmed that the transfection was successful (Fig. 6A). The viability, colony formation ability, apoptosis, migration, and invasion of the transfected cells were determined, and it was found that the downregulation of CRSIP3 repressed viability, colony formation ability, migration, and invasion of LC cells, and facilitated the LC cell apoptosis (Fig. 6B–F). These results suggest that downregulated CRISP3 suppresses the malignancy of LC cells.

### LINC01342 promotes LC cell development through the miR-508-5p/CRISP3 axis

**Fig. 7:**
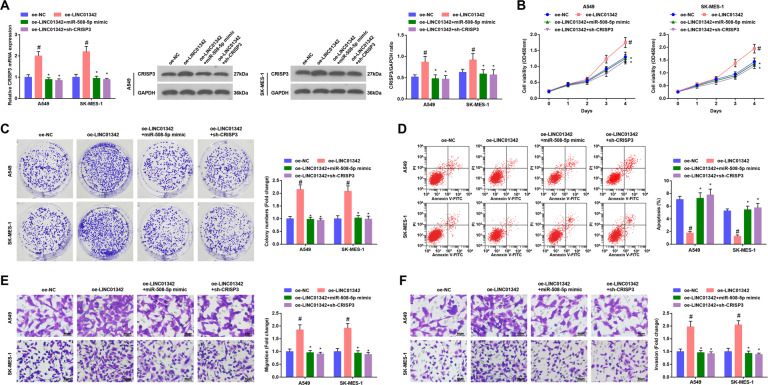
LINC01342 promotes LC cell development through the miR-508-5p/CRISP3 axis. **A** CRISP3 mRNA and protein expression in LC cells was detected using RT-qPCR and Western blot analysis. **B** Cell viability was assessed using CCK-8 assay. **C** Colony formation ability of cells was detected using cell colony formation assay. **D** Apoptosis of cells was assessed using flow cytometry. **E** and **F** Migration and invasion of cells were detected using Transwell assay; #*P* < 0.05 vs. the oe-NC group; *P < 0.05 vs. the oe-LINC01342 group.

The rescue experiment was performed to explore whether LINC01342 could facilitate LC cell development through the miR-508-5p/CRISP3 axis. It was confirmed through RT-qPCR and Western blot analysis that the miR-508-5p mimic and sh-CRISP3 were successfully transfected into LC cells expressing LINC01342 (Fig. 7A). The biological functions of transfected cells were further analyzed and we observed that overexpression of LINC01342 enhanced the growth of LC cells, while both miR-508-5p elevation and CRISP3 inhibition reversed the effects of LINC01342 overexpression on LC cells in vitro (Fig. 7B–F).

## Discussion

LC is the main cause of cancer-related mortality, contributing to ~25.3% of all cancer deaths, with a poor prognosis [19]. This study was performed to investigate the role of the LINC01342/miR-508-5p/CRISP3 axis in LC, and our results indicated that LINC01342 promoted the malignant behaviors of LC cells through regulating miR-508-5p/CRISP3, thus contributing to LC progression.

To begin with, we detected the expression of LINC01342 expression in tissues and cells and found that it was upregulated in LC tissues and cells, respectively, compared with adjacent normal tissues and normal bronchial epithelial cells 16HBE. Due to the limited literature reporting LINC01342, we can only find in a recent study that LINC01342 expression was increased in the OC tissue samples [9]. Considering this aberrant upregulation of LINC01342, we analyzed its role in the prognosis of LC patients. The patients were divided into the high and low LINC01342 expression groups based on the median value of LINC01342 expression to detect the association between LINC01342 expression and clinicopathological characteristics of LC patients. It was found in our findings that LC patients with high LINC01342 expression had a later TNM stage and severer LNM in contrast to those with low LINC01342 expression. Moreover, we also found that LINC01342 upregulation indicated a poorer survival rate in LC patients. Thus, we concluded that LINC01342 functioned as an oncogene in LC. Moreover, we knocked LINC0134 down in LC cells to observe its role in cancer cell growth. Through the CCK-8 assay, colony formation assay, flow cytometry, scratch test, and Transwell assay that the knockdown of LINC01342 inhibited proliferation, migration, and invasion, and inhibited apoptosis of LC cells. Similarly, Zhang et al. have revealed that downregulation of LINC01342 repressed the proliferative and metastatic abilities of OC cells [9]. The identification of LINC01342 in human cancer cell biological functions demands further explorations.

As predicted through a bioinformatic website, there existed a binding region between LINC01342 and miR-508-5p. Furthermore, this binding relationship between LINC01342 and miR-508-5p was confirmed using dual-luciferase reporter gene assay and RIP assay. MiR-508-5p expression was also detected in our research and we found that it was downregulated in LC tissues and cell lines. Consistently, Liu et al. have reported that the expression of miR-508-5p was decreased in glioma tissues and cell lines [20], and it has been demonstrated as well that miR-508-5p was downregulated in hepatocellular carcinoma tissues versus the non-tumorous tissues [21]. These data revealed the downregulation of miR-508-5p in human cancers. Therefore, we regulated miR-508-5p expression in LC cells to assess its role during LC cell growth. It was found through a series of essays in our study that the elevation of miR-508-5p inhibited the malignant episodes of LC cells in vivo and in vitro. Similar to our findings, a study has proposed that miR-508-5p upregulation markedly suppressed the proliferation, migration, and invasion of melanoma cells [13], and miR-508-5p has been found to reduce glioma cell growth as well [22]. Furthermore, we found through the bioinformatic analysis and confirmed using dual-luciferase reporter gene assay that CRISP3 was a target gene of mIR-508-5p. Herein, we detected the expression of CRISP3 and assessed its role in LC cells. It was found in our results that CRISP3 was oe in LC cells and its downregulation inhibited the LC cell growth. In accordance with our findings, Chen et al. have found that that CRISP3 was upregulated in NSCLC patients after chemotherapy [17]. Furthermore, a publication has elucidated that CRISP3 facilitated cell motility and invasion in prostate cancer [16]. The low expression of CRISP3 has been reported to predict a favorable prognosis in patients with mammary carcinoma, and also weakened the migration and invasion of mammary carcinoma cells [23].

## Conclusion

In conclusion, we found that the knockdown of LINC01342 upregulates miR-508-5p to inhibit the progression of LC through the inhibition of CRISP3. This study may provide novel biomarkers for LC prognosis and treatment. However, the detailed mechanism was not ulteriorly explored in this study due to limited conditions, and we would perform further studies in the future.

## Materials and methods

### Ethics statement

Written informed consent was acquired from all patients before this study. The protocol of this study was confirmed by the Ethics Committee of The First Hospital of China Medical University and based on the ethical principles for medical research involving human subjects of the Helsinki Declaration. Animal experiments were strictly in accordance with the Guide to the Management and Use of Laboratory Animals issued by the National Institutes of Health. The protocol of animal experiments was approved by the Institutional Animal Care and Use Committee of The First Hospital of China Medical University.

### Study subjects

One hundred and six pairs of primary LC tissues and matched adjacent normal tissues were harvested from patients (70 males and 36 females, ages 26–75 years) accepted surgical resection in The First Hospital of China Medical University, and all of the cancer tissues were confirmed by pathological diagnosis.

### Cell culture

LC cell lines (A549, PC-9, SK-MES-1, H1299, and H1975) and normal bronchial epithelial cell line 16HBE (Shanghai Institute of Biochemistry and Cell Biology, Chinese Academy of Sciences, Shanghai, China) were incubated in Dulbecco’s modified Eagle medium (DMEM, Qiangyao Technology, Guangdong, China) containing 10% fetal bovine serum (FBS, Gibco, CA, USA), 100 units/mL penicillin (Haoo, Heilongjiang, China) and 100 μg/mL streptomycin (Ruiyang Technology Co., Ltd., Beijing, China).

### Cell transfection and grouping

A549 and SK-MES-1 cells were seeded onto six-well plates and transfected with oligonucleotides or plasmids using Lipofectamine 2000 (Invitrogen, CA, USA). The cells were classified into: the small interfering RNA (si)-negative control (NC) group (transfection of NC si-RNA), si-LINC01342 group (transfection of si-RNA-LINC01342), mimic NC group (transfection of miR-508-5p mimic NC), miR-508-5p mimic (transfection of miR-508-5p mimic), short hairpin RNA (sh)-NC group (transfection of sh-RNA NC), sh-CRISP3 group (transfection of CRISP3 sh-RNA), overexpressed (oe)-NC group (transfection of unrelated LINC01342 plasmid), oe-LINC01342 group (transfection of plasmid overexpressing LINC01342), oe-LINC01342 + miR-508-5p mimic group (transfection of plasmid overexpressing LINC01342 and miR-508-5p mimic) and oe-LINC01342 + sh-CRISP3 group (transfection of plasmid overexpressing LINC01342 and CRISP3 sh-RNA). MiR-508-5p mimic and its NC were acquired from RiboBio Co., Ltd. (Guangdong, China); si-LINC01342, oe-NC, oe-LINC01342, and si-NC were purchased from GenePharma Co., Ltd. (Shanghai, China); sh-CRISP3 and sh-NC were obtained from Sangon Biotechnology Co., Ltd. (Shanghai, China).

### Cell counting kit-8 (CCK-8) assay

According to manufactures’ information, the cell proliferation was determined using CCK-8 kits (Dojindo, Shanghai, China). Cells were plated onto 96-well plates and incubated for 0–4 days. A microplate reader was used to detect the absorbance of cells at 450 nm, and then the proliferation rate was calculated [24].

### Colony formation assay

Cells that had been transfected for 24 h were seeded onto a six-well plate at 1000 cells/well for 14 days culture. Then, cells were fixed and stained with 0.5% crystal violet dye solution. The colonies (a colony contains over 50 cells) were counted under a microscope [25].

### Flow cytometry

The apoptosis of cells was determined using the fluorescein isothiocyanate apoptosis detection kits (BioLegend, CA, USA) referring to a publication [26].

### Transwell assay

The migration and invasion of cells were determined using Transwell chambers (BD Biosciences, NJ, USA) as previously described [27]. Matrigel was used in the invasion assay but not the migration assay.

### Reverse transcription-quantitative polymerase chain reaction (RT-qPCR)

Total RNA was extracted using Trizol kits (Invitrogen, CA, USA) and reversely transcribed into cDNA. The SYBR Green PCR Kit (Takara) was used for the quantification analysis. U6 and glyceraldehyde phosphate dehydrogenase (GAPDH) were used as internal references. Data were analyzed using 2^−ΔΔCt^ method and primer sequences (RiboBio) were shown in Table 2.

**Table 2:** Primer sequence.

Gene	Sequence (5′ → 3′)
LINC01342	F: GTTTGACTTGTTCAGGCACA
	R: GTCCTCCAAAGACGAGAACAG
miR-508-5p	F: TACTCCAGAGGGCGTCACTCATG
CRISP3	F: AAATCATGGAAAATAAGGGAATCCT
	R: CCAAGAAGCACATTGCATTTG
U6	F: CTCGCTTCGGCAGCACA
	R: AACGCTTCACGAATTTGCGT
GAPDH	F: CATCCATGACAACTTTGGTATCGT
	R: CCATCACGCCACAGTTTCC

F forward, R reverse, miR-508-5p microRNA-508-5p, GAPDH glyceraldehyde phosphate dehydrogenase.

### Western blot analysis

Total protein in tissues or cells was extracted. The proteins were conducted with gel electrophoresis, transferred onto membranes, and incubated with primary antibodies CRISP3 (1:1000, C9996, Sigma-Aldrich, MI, USA) and GAPDH (1:1000, ab8245, Abcam, CA, USA), and then were incubated with relative secondary antibody for 1 h. Subsequently, the bands were visualized using the enhanced chemiluminescent detection system (Bio-Rad, CA, USA) and the gray value was determined.

### Dual-luciferase reporter gene assay

The fragments of LINC01342 wild type (WT), LINC01342 mutant type (MUT), CRISP3-WT, and CRISP3-MUT containing miR-508-5p-binding site were designed by GenePharma. According to the instructions, the pmir-GLO vector was used to insert the fragments into the luciferase reporter molecule. A549 and SK-MES-1 cells were seeded in six-well plates and transfected with miR-508-5p mimic or mimic NC. Then, a total of 100 ng pmir-GLO reporter vector containing each fragment was transfected into A549 and SK-MES-1 cells. After transfection, the cells were incubated at 37 °C and with 5% CO_2_. The luciferase activity was determined by a dual-luciferase reporter gene detection system (Promega, WI, USA).

### RNA immunoprecipitation (RIP) assay

According to the manufacturer’s information, Magna RIP^TM^ RNA-binding protein immunoprecipitation Kit (Millipore Inc., MA, USA) and the Ago2 antibody (Abcam) or immunoglobulin G (IgG) antibody (the NC) were used for RIP assay. The immunoprecipitated RNA was confirmed by RT-qPCR.

### Subcutaneous tumorigenesis in nude mice

BALB/c nude mice aging 4–6 weeks were subcutaneously injected with A549 or SK-MES-1 cells that had been stably transfected with si-LINC01342 or si-NC. Each group contained five nude mice. The longest and shortest diameters of xenografts were recorded with a caliper every week, and the tumor volume was calculated (volume = 0.5 × length × width^2^). Injected for 28 days, all mice were euthanized with the xenografts collected and weighed [24].

### Statistical analysis

All data analyses were conducted using SPSS 21.0 software (IBM Corp., Armonk, NY, USA). The measurement data were expressed as mean ± standard deviation. The *t*-test was performed for comparisons between two groups and one-way analysis of variance (ANOVA) was used for comparisons among multiple groups, followed by Tukey’s post hoc test. Correlations among expression of LINC01342, miR-508-5p, and CRIPS3 were analyzed using the Pearson test. *P* value < 0.05 was indicative of statistically significant difference.

## Supplementary information

supplementary Figure 1

supplementary Figure 1、Table

cddiscovery-author-contribution-form
