# Supplementary figures and images for: LINC01342 silencing upregulates microRNA-508-5p to inhibit progression of lung cancer by reducing cysteine-rich secretory protein 3
Source: Cell Death Discov. 2021 Sep 9;7:238. doi: 10.1038/s41420-021-00613-x (PMC8429695; doi:10.1038/s41420-021-00613-x)

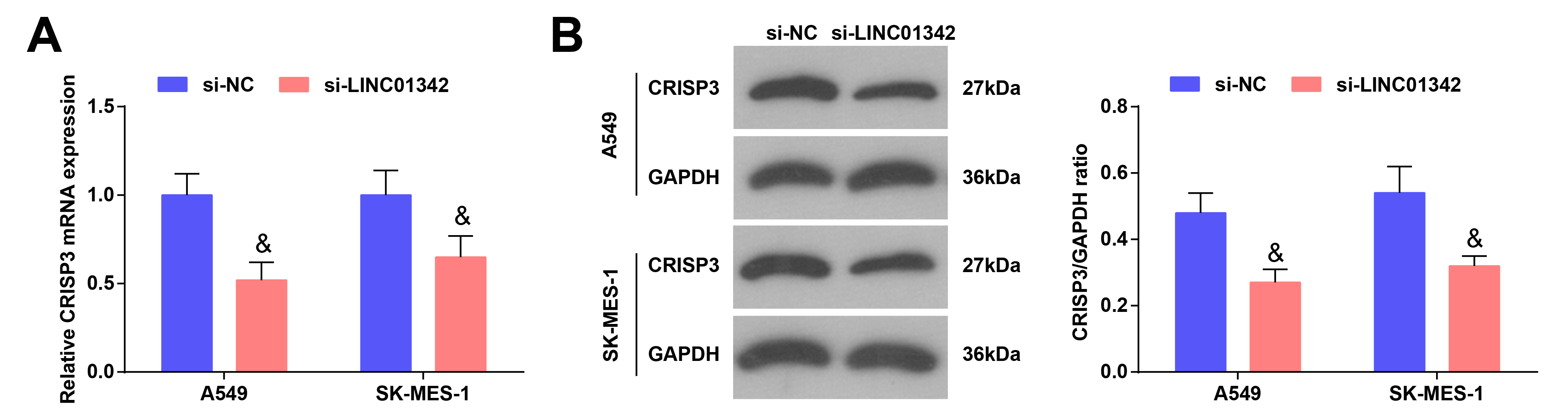

Supplement: Supplementary file 1 — supplementary Figure 1 [file 41420_2021_613_MOESM1_ESM.tif]
