# Supplementary material for: LINC01342 silencing upregulates microRNA-508-5p to inhibit progression of lung cancer by reducing cysteine-rich secretory protein 3
Source: Cell Death Discov. 2021 Sep 9;7:238. doi: 10.1038/s41420-021-00613-x (PMC8429695; doi:10.1038/s41420-021-00613-x)
Supplement: Supplementary file 2 — supplementary Figure 1、Table [file 41420_2021_613_MOESM2_ESM.docx]

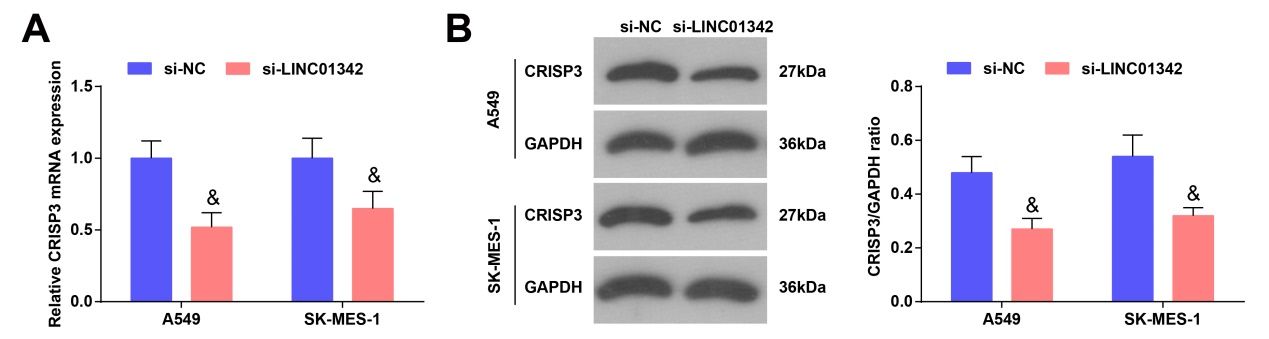


**Table 1** Primer sequence

| Gene | Sequence (5’→3’) |
| --- | --- |
| LINC01342 | F: GTTTGACTTGTTCAGGCACA |
|  | R: GTCCTCCAAAGACGAGAACAG |
| miR-508-5p | F: ACACTCCAGCTGGGTACTCCAGAGGGCGTCACT |
|  | R: TGGTGTCGTGGAGTCG |
| CRISP3 | F: GCACCTTCCTTCTGTCA |
|  | R: CAGCCTCTTTGTTCCATT |
| U6 | F: TGCGGGTGCTCGCTTCGGCAGC |
|  | R: CCAGTGCAGGGTCCGAGGT |
| GAPDH | F: GAAGGTGAAGGTCGGAGTC |
|  | R: GAAGATGGTGATGGGATTTC |

Note: F, forward; R, reverse; miR-508-5p, microRNA-508-5p; GAPDH, glyceraldehyde phosphate dehydrogenase.

**Table 2** Relationship between LINC01342 expression and clinicopathological characteristics of lung cancer patients

| **Parameter** | **Case** | | **LINC01342** | | | | **P value** |
| --- | --- | --- | --- | --- | --- | --- | --- |
|  |  |  | **Low expression**  **(n = 52)** | | | **High expression**  **(n = 54)** |  |
| Age (years) | | | | | | | 0.567 |
| < 60 | 54 | | 28 | | | 26 |  |
| ≥ 60 | 52 | | 24 | | | 28 |  |
| Gender | | | | | | | 0.686 |
| Male | 68 | | 32 | | | 36 |  |
| Female | 38 | | 20 | | | 18 |  |
| Smoking history | | | | | | | 0.846 |
| Yes | | 57 | | 27 | 30 | |  |
| No | | 49 | | 25 | 24 | |  |
| Tumor size | | | | | | | 0.340 |
| ≤ 3 cm | 54 | | 29 | | | 25 |  |
| > 3 cm | 52 | | 23 | | | 29 |  |
| Histology type | | | | | | | 0.234 |
| Adenocarinoma | 64 | | 28 | | | 36 |  |
| Squamous | 42 | | 24 | | | 18 |  |
| Lymph nodes metastasis | | | | | | | 0.034 |
| No | 74 | | 31 | | | 43 |  |
| Yes | 32 | | 21 | | | 11 |  |
| TNM Stage | | | | | | | 0.025 |
| I - II | 69 | | 28 | | | 41 |  |
| III-IV | 37 | | 24 | | | 13 |  |

Note: TNM, tumor-node-metastasis. The enumeration data were analyzed using chi-square test.
