# Supplementary material for: LINC01342 silencing upregulates microRNA-508-5p to inhibit progression of lung cancer by reducing cysteine-rich secretory protein 3
Source: Cell Death Discov. 2021 Sep 9;7:238. doi: 10.1038/s41420-021-00613-x (PMC8429695; doi:10.1038/s41420-021-00613-x)
Supplement: Supplementary file 3 — cddiscovery-author-contribution-form [file 41420_2021_613_MOESM3_ESM.pdf]

DECLARATION OF CONTRIBUTIONS TO ARTICLE

ADMC

Manuscript Number: CDDISCOVERY-21-1593

Journal Name: *Cell Death Discovery* (the 'Journal')

Proposed Title of the Contribution: author contributions (the 'Contribution)

Author(s): Qiming Shen, Zhe Xu, Guanghao Sun, Haoyou Wang, Lin Zhang (the 'Authors')

For all *CDDis*covery articles, each person named as an author in the published version must be able to show he or she has contributed substantially to the article.

Authorship credit should be based on 1) substantial contributions to conception and design, acquisition of data, or analysis and interpretation of data; 2) drafting the article or revising it critically for important intellectual content; and 3) final approval of the version to be published. Authors should meet conditions 1, 2 and 3.

Any person who cannot be shown to have made a substantial contribution to the article cannot be listed as an author in the final version. The name of any person who is deemed to have made a minor contribution can, however, appear in the Acknowledgments section of the article.

Please complete the table below to indicate the contributions of all named authors to the manuscript.

Author Full Name:	Specification of Contribution to the Manuscript:
Zhe Xu and Guanghao Sun	data analysis
Qiming Shen	study design
Haoyou Wang and Lin Zhang	experimental studies
Qiming Shen	manuscript editing

Please complete the table below to indicate the contributions of all named authors to the figures.

Figure 1:

Qiming Shen, Zhe Xu, Guanghao Sun, Haoyou Wang, Lin Zhang

Figure 2:

Qiming Shen, Zhe Xu, Guanghao Sun, Haoyou Wang, Lin Zhang

Figure 3:

Qiming Shen, Zhe Xu, Guanghao Sun, Haoyou Wang, Lin Zhang

Figure 4:

Qiming Shen, Zhe Xu, Guanghao Sun, Haoyou Wang, Lin Zhang

Figure 5:

Qiming Shen, Zhe Xu, Guanghao Sun, Haoyou Wang, Lin Zhang

Figure 6:

Qiming Shen, Zhe Xu, Guanghao Sun, Haoyou Wang, Lin Zhang

Signed for and on behalf of the Author(s):	Print Name:	Date:
Qiming Shen	Qiming Shen	2021.5.27
